# Effect of type 2 diabetes on coronary artery ectasia: smaller lesion diameter and shorter lesion length but similar adverse cardiovascular events

**DOI:** 10.1186/s12933-022-01444-5

**Published:** 2022-01-19

**Authors:** Zhongxing Cai, Luqi Li, Haoyu Wang, Sheng Yuan, Dong Yin, Weihua Song, Kefei Dou

**Affiliations:** 1 Cardiometabolic Medicine Center, Department of Cardiology, Fuwai Hospital, National Center for Cardiovascular Diseases, Chinese Academy of Medical Sciences and Peking Union Medical College, No.167, Beilishi Road, Xicheng District, Beijing, 100037 China; 2 State Key Laboratory of Cardiovascular Disease, Beijing, China; 3 Institute of Medical Information, Chinese Academy of Medical Sciences and Peking Union Medical College, Beijing, China

**Keywords:** Diabetes mellitus, Coronary artery ectasia, Clinical outcomes

## Abstract

**Background:**

Coronary artery ectasia (CAE) is a rare finding in coronary angiography and associated with poor clinical outcomes. Unlike atherosclerosis, diabetes mellitus (DM) is not commonly associated with CAE. This study aims to investigate the effect of type 2 diabetes mellitus (DM2) on coronary artery ectasia, especially the differences in angiographic characteristics and clinical outcomes.

**Methods:**

Patients with angiographically confirmed CAE from 2009 to 2015 were included. Quantitative coronary angiography (QCA) was performed to measure the diameter and length of the dilated lesion. The primary endpoint was the maximum diameter and maximum length of the dilated lesion at baseline coronary angiography. The secondary endpoint was 5-year major adverse cardiovascular events (MACE), which was a component of cardiovascular death and nonfatal myocardial infarction (MI). Propensity score weighting (PSW) and propensity score matching (PSM) were used to balance covariates. Kaplan–Meier method and Cox regression were performed to assess the clinical outcomes.

**Results:**

A total of 1128 patients were included and 258 were combined with DM2. In the DM2 group, the maximum diameter of dilated lesion was significantly lower (5.26 mm vs. 5.47 mm, P  = 0.004) and the maximum length of the dilated lesion was significantly shorter (25.20 mm vs. 31.34 mm, P  = 0.002). This reduction in dilated lesion diameter (5.26 mm vs. 5.41 mm, P  = 0.050 in PSW; 5.26 mm vs. 5.46 mm, P  = 0.007 in PSM, respectively) and length (25.17 mm vs. 30.17 mm, P  = 0.010 in PSW; 25.20 mm vs. 30.81 mm, P  = 0.012 in PSM, respectively) was consistently observed in the propensity score analysis. A total of 27 cardiovascular deaths and 41 myocardial infarctions occurred at 5-year follow-up. Compared with non-DM group, there were similar risks of MACE (6.02% vs. 6.27%; HR 0.96, 95% CI 0.54–1.71, P  = 0.894), cardiovascular death (2.05% vs. 2.61%; HR 0.78, 95% CI 0.29–2.05, P  = 0.605) and MI (4.07% vs. 3.72%; HR 1.11, 95% CI 0.54–2.26, P  = 0.782) in patients with DM2. Consistent result was observed in multivariable regression.

**Conclusions:**

Compared to non-DM patients, patients with CAE and type 2 diabetes were associated with a smaller diameter and shorter length of dilated vessels, suggesting the important effect of DM2 on the pathophysiological process of CAE. Similar risks of MACE were found during 5-year follow up among diabetic and non-DM patients.

**Supplementary Information:**

The online version contains supplementary material available at 10.1186/s12933-022-01444-5.

## Background

Coronary artery ectasia (CAE) is defined as abnormal coronary artery dilation of at least 1.5 times the adjacent normal segment [1]. It is found in 0.3–5% of coronary angiography [2]. CAE used to be considered as a variant of coronary atherosclerosis [3], but later several studies indicated poor clinical outcomes in patients with CAE, and increased risk of adverse cardiovascular events was observed [4–6]. Besides, the association of CAE and other cardiovascular diseases including aortic aneurysms and varicose veins has been reported [7, 8], indicating the complex mechanisms of CAE.

In contrast to atherosclerosis, diabetes mellitus (DM) was not commonly associated with CAE. The prevalence of diabetes mellitus ranged from 6.9 to 29% in different studies, and a consistently inverse association between diabetes mellitus and CAE was observed [5, 6]. This negative relationship was consistent with the findings in abdominal and thoracic aortic aneurysms [9]. And several studies showed diabetic patients developed smaller aortic aneurysms compared to non-diabetic individuals [10, 11]. However, the effect of diabetes mellitus on CAE has not been studied. The differences between CAE patients with and without diabetes mellitus remained unknown.

This study aimed to investigate the effect of type 2 diabetes mellitus (DM2) on coronary artery ectasia, especially the differences in angiographic characteristics and clinical outcomes among patients with and without diabetes.

## Methods

### Study population

Consecutive patients with angiographically confirmed CAE between 2009 and 2015 in Fuwai hospital were included and divided into DM2 group and non-DM group. All patients underwent coronary angiography for suspected ischemic heart disease. The angiographic criteria of CAE were defined as: (1) abnormal dilation of more than 1.5-fold the diameter of adjacent normal segments; or (2) if there was no adjacent normal segment found, normal reference values of corresponding segments from data in age-sex matched patients with normal coronary angiography were used as reference diameters, and CAE was defined as abnormal dilation of more than 1.5-fold the reference diameter as previously reported (Additional file 1: Table S1) [5, 12]. The diagnostic criteria for diabetes were as follows [13]: (1) Typical symptoms of diabetes (polydipsia, polyuria, polyphagia, and weight loss) plus random blood glucose testing  ≥ 11.1 mmol/l (200 mg/dl); or (2) Fasting plasma glucose  ≥ 7 mmol/l (126 mg/dl); or (3) 2 h-blood glucose testing  ≥ 11.1 mmol/l (200 mg/dl) after the glucose load test; or (4) diagnosed with diabetes and being treated with hypoglycemic drugs or insulin. The exclusion criteria were (1) insignificant dilated vessel diameter which was less than 1.5 times the reference diameter; (2) coronary artery fistula; (3) stent-related coronary artery aneurysms; (4) known autoimmune disease; (5) missing imaging files in Digital Imaging and Communications in Medicine (DICOM) format; (6) valvular heart disease; or (7) history of coronary artery bypass grafting (CABG). The angiogram of each patient was screened by 2 experienced interventional cardiologists.

This study was approved by the Ethics Committee of Fuwai Hospital.

### Clinical data collection

Medical record including medical history, laboratory test and echocardiography results were obtained from the hospital’s electronic medical records system. The modified Modification of Diet in Renal Disease (MDRD) equations based on Chinese patients were applied to calculate the estimated glomerular filtration rate (eGFR) [14]. Laboratory test results were at baseline and before coronary angiography.

### Angiographic evaluation and quantitative coronary angiography (QCA)

The DICOM format files of baseline coronary angiography were analyzed with Qangio XA version 7.3 (Medis, Leiden, Netherlands) by an independent catheterization core laboratory. Each dilated lesion was measured for lesion diameter, reference diameter, lesion length and vessel length. Then the maximum diameter of the dilated lesion and maximum length of dilated lesion of each patient were calculated. Patients with dilated lesion involved ≥ 1/3 of the coronary vessel were classified as diffuse CAE and those involved < 1/3 were focal CAE or aneurysms [15, 16]. Maximum diameter  ≥ 5 mm was classified as large and maximum diameter  < 5 mm was defined as small. Markis classification of CAE was assessed [17]. Diffuse ectasia of 2 or 3 vessels was classified as type I, diffuse disease in 1 vessel and localized disease in another vessel as type II, diffuse ectasia of 1 vessel only as type III, and localized or segmental ectasia as type IV. Coronary artery disease (CAD) was defined as at least 50% diameter stenosis of the left main coronary artery, the left anterior descending artery (LAD), the left circumflex (LCX) coronary artery, the right coronary artery (RCA), or the main branch of the coronary vessels with diameter  > 2 mm. The synergy between PCI with Taxus and cardiac Surgery (SYNTAX) scores of each patient were calculated to quantify the severity of combined coronary artery disease [18].

### Endpoints and follow-up

The primary endpoint was the maximum diameter and maximum length of the dilated lesion at baseline coronary angiography. The secondary endpoint was 5-year major adverse cardiovascular events (MACE), which was a component of cardiovascular death and nonfatal myocardial infarction (MI). Follow-up was conducted annually by telephone interviewers using standardized questionnaires.

### Statistical analysis

Normally distributed continuous variables were expressed as mean  ±  standard deviation and compared using the t test. Continuous data with non-normal distribution were summarized as median (interquartile range, IQR) and compared using the Mann–Whitney test. Categorical variables were expressed as counts (composition ratio), and compared using the Chi-square test or Fisher exact test as appropriate. Propensity score matching (PSM), propensity score weighting (PSW) and subgroup analysis were performed as sensitivity analysis to adjust covariates. We used a multivariable logistic regression model to estimate propensity scores (PS), with DM2 group as the dependent variable and the following variables as covariates: age, gender, body mass index (BMI), hypertension, dyslipidemia, peripheral arterial disease, smoking status, acute MI, previous MI, previous PCI, family history of CAD, LVEF and eGFR. These variables were chosen either because of significant differences in baseline characteristics between groups or the potential relevance with coronary artery diameters. Propensity score weighting was performed using standardized mortality ratio weighting (SMRW). PS Matching was performed using the optimal pair matching with a 1:2 ratio. Standardized difference less than 0.1 indicated a good balance after the PS method. Survival analysis was performed using the Kaplan–Meier method and comparisons between the 2 groups were applied by the log-rank test. Cox proportional hazards regression was conducted to access hazard ratio and multivariable Cox regression was applied to control potential confounding. Two-tailed P value  < 0.05 was regarded as statistical significance in this study. All analyses were performed by R 4.1.0 (R Foundation for Statistical Computing, Vienna, Austria).

## Results

### Clinical features and characteristics

**Fig. 1:**
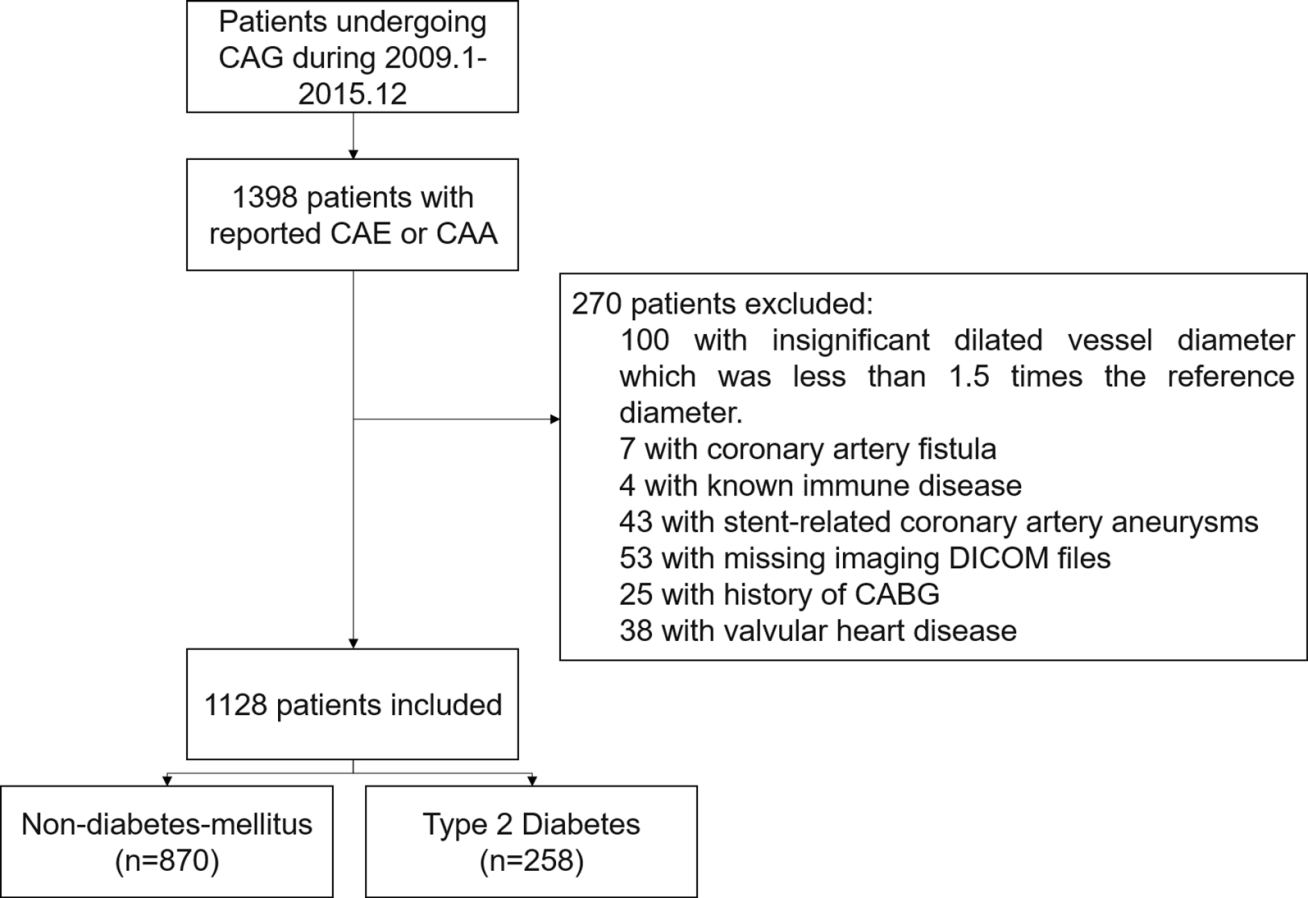
Flowchart of the study population

The incidence of CAE in the procedure reports was 1.08%. A total of 1128 consecutive patients, of which 870 were non-diabetic, were included in this study and the flowchart of the study objects was shown in Fig. 1. All the 258 diabetic patients were type 2 DM and the median duration of DM diagnosis is 6 years (IQR 2–10 years). There are several differences between the baseline characteristics of the DM2 group and non-DM group. Patients with CAE combined with DM2 were significantly older, and with a significantly higher incidence of dyslipidemia. Slightly higher BMI was also observed in DM2 group. And acute MI was less common in the DM2 group. 213 patients were treated with glucose-lowering drugs or insulin before admission in the DM2 group, and details of the baseline characteristic of the study objects were shown in Table 1.

**Table 1:** Baseline characteristics of the study population

	Non-DM group	DM2 group	P
Patients no.	870	258	
Male	743 (85.4)	208 (80.6)	0.079
Age, years	56.40 ± 11.24	59.80 ± 9.72	< 0.001
Height, cm	170.00 [166.00, 175.00]	170.00 [164.00, 175.00]	0.119
Weight, kg	76.00 [69.00, 85.00]	77.00 [68.25, 85.00]	0.881
BMI	26.33 [24.22, 28.73]	27.01 [24.52, 29.38]	0.093
Clinical presence (%)	–	–	0.037
Asymptomatic	18 (2.1)	6 (2.3)	
Stable angina	278 (32.0)	90 (34.9)	
Unstable angina	384 (44.1)	125 (48.4)	
NSTEMI	54 (6.2)	13 (5.0)	
STEMI	122 (14.0)	19 (7.4)	
Dyspnea	7 (0.8)	0 (0.0)	
Palpitation	7 (0.8)	5 (1.9)	
Acute MI	176 (20.2)	32 (12.4)	0.006
Prior MI	244 (28.0)	66 (25.6)	0.484
Prior PCI	203 (23.3)	71 (27.5)	0.196
Hypertension	555 (63.8)	182 (70.5)	0.054
Dyslipidemia	536 (61.6)	183 (70.9)	0.008
Peripheral arterial disease	60 (6.9)	21 (8.1)	0.588
Family history of CAD	145 (16.7)	44 (17.1)	0.959
Current smoker	306 (35.2)	88 (34.1)	0.810
LVEF	62.00 [57.00, 66.20]	62.05 [58.02, 66.10]	0.417
LVID	50.00 [47.00, 53.00]	50.00 [46.00, 53.00]	0.461
HbA1c	5.90 [5.60, 6.10]	7.30 [6.50, 8.10]	< 0.001
Cr	78.30 [69.14, 88.50]	76.10 [67.14, 86.06]	0.041
EGFR	114.10 ± 25.51	114.49 ± 28.33	0.832
HsCRP	1.91 [0.98, 4.29]	1.91 [1.09, 4.38]	0.531
TC	4.23 [3.56, 5.00]	4.05 [3.41, 4.72]	0.015
TG	1.58 [1.14, 2.09]	1.70 [1.27, 2.26]	0.006
LDL	2.50 [1.94, 3.16]	2.38 [1.84, 2.94]	0.018
HDL	0.98 [0.85, 1.16]	0.95 [0.79, 1.09]	0.001
NT-proBNP	205.73 [109.25, 269.00]	196.26 [109.25, 238.24]	0.307
Medications at discharge	–	–	–
Aspirin	843 (96.9)	253 (98.1)	0.437
Clopidogrel	586 (67.4)	178 (69.0)	0.676
Ticagrelor	22 (2.5)	6 (2.3)	1.000
Statins	786 (90.3)	236 (91.5)	0.672
ACEI/ARB	472 (54.3)	149 (57.8)	0.357
β-blocker	736 (84.6)	225 (87.2)	0.348
CCB_DHP	194 (22.3)	81 (31.4)	0.004
CCB_nonDHP	160 (18.4)	53 (20.5)	0.493
Nitrates	730 (83.9)	227 (88.0)	0.132
Revascularization (%)	–	–	1.000
None	361 (41.5)	107 (41.5)	–
PCI	377 (43.3)	112 (43.4)	–
CABG	132 (15.2)	39 (15.1)	–
Duration of diabetes diagnosis, years	–	6.00 [2.00, 10.00]	–
Glucose-lowering treatment before admission	–	213 (82.56)	–
Insulin	–	89 (34.50)	–
Metformin	–	68 (26.36)	–
α-glucosidase	–	95 (36.82)	–
Sulfonylurea	–	46 (17.83)	–
Thiazolidinedione	–	4 (1.55)	–
Glinides	–	19 (7.36)	–
DPP-4 inhibitor	–	3 (1.16)	–
GLP-1RA	–	0 (0.00)	–
Glucose-lowering treatment at discharge	–	228 (83.37)	–
Insulin	–	96 (37.21)	–
Metformin	–	78 (30.23)	–
α-glucosidase	–	115 (44.57)	–
Sulfonylurea	–	49 (18.99)	–
Thiazolidinedione	–	4 (1.55)	–
Glinides	–	18 (6.98)	–
DPP-4 inhibitor	–	5 (1.94)	–
GLP-1RA	–	2 (0.78)	–

Values are mean  ±  SD, n (%), or median (interquartile range) unless otherwise stated

*ACEI *angiotensin-converting enzyme inhibitors; *ARB *angiotensin receptor blockers; *BMI *body mass index; *CABG *coronary artery bypass grafting; *CAD *coronary artery disease; *DHP *dihydropyridine; *DPP-4 *dipeptidyl peptidase-4; *EGFR *estimated glomerular filtration rate; *GLP-1RA *glucagon-like peptide-1 receptor agonist; *HsCRP *high sensitivity C-reactive protein; *LVEF *left ventricular ejection fraction; *LVID *left ventricular internal dimension; *MI *myocardial infarction; *NSTEMI *non–ST-segment elevation myocardial infarction; *PCI *percutaneous coronary intervention; *STEMI *ST-segment elevation myocardial infarction

### Angiographic evaluation and QCA analysis

The location of dilated vessels was similar among the 2 groups. The right coronary artery was the most common dilated vessel, followed by the left anterior descending and left circumflex artery. However, the DM2 group was associated with more coronary artery disease and a slightly higher SYNTAX score. And focal CAE seemed more likely found in the DM2 group but it didn’t reach statistically significant. Detailed angiographic characteristics of the study objects were shown in Table 2.

**Table 2:** Angiographical characteristics of the study population

	Non-DM group	DM2 group	P
Patient no.	870	258	
Combined CAD (%)			0.052
None	102 (11.7)	15 (5.8)	
Single vessel	173 (19.9)	43 (16.7)	
Double vessels	222 (25.5)	78 (30.2)	
Three vessels	295 (33.9)	99 (38.4)	
LM only	3 (0.3)	0 (0.0)	
LM + single vessel	6 (0.7)	2 (0.8)	
LM + double vessels	13 (1.5)	1 (0.4)	
LM + three vessels	56 (6.4)	20 (7.8)	
SYNTAX score	14.00 [7.00, 21.00]	15.50 [8.25, 22.00]	0.048
SYNTAX score level (%)			0.376
Low (≤ 22)	668 (76.8)	195 (75.6)	
Mid (> 22 and ≤ 32)	166 (19.1)	47 (18.2)	
High (> 32)	36 (4.1)	16 (6.2)	
LM ectasia	108 (12.4)	23 (8.9)	0.153
LAD ectasia	363 (41.7)	110 (42.6)	0.85
LCX ectasia	333 (38.3)	97 (37.6)	0.901
RCA ectasia	540 (62.1)	146 (56.6)	0.131
Markis classification			0.06
Type I	63 (7.2)	9 (3.5)	
Type II	185 (21.3)	59 (22.9)	
Type III	223 (25.6)	56 (21.7)	
Type IV	399 (45.9)	134 (51.9)	
Diffuse dilation	472 (54.3)	124 (48.1)	0.093
Focal dilation (aneurysms)	398 (45.7)	134 (51.9%)	0.093
Big CAE (max diameter > 5 mm)	577 (66.3)	147 (57.0)	0.007
Contrast agent stasis	236 (27.1)	56 (21.7)	0.096
Thrombus in dilated segment	16 (1.8)	4 (1.6)	0.968
Calcification in dilated segment	52 (6.0)	23 (8.9)	0.128
Max. lesion diameter	5.47 [4.67, 6.29]	5.26 [4.46, 5.98]	0.004
Max. lesion length	31.34 [13.32, 62.97]	25.20 [10.39, 46.76]	0.002

Values are mean  ±  SD, n (%), or median [interquartile range] unless otherwise stated

*CAD *coronary artery disease; *CAE *coronary artery ectasia; *LAD *left anterior descending artery; *LCX *left circumflex artery; *LM *left main; *RCA *right coronary artery; *SYNTAX *synergy between PCI with *Taxus* and Cardiac Surgery

**Fig. 2:**
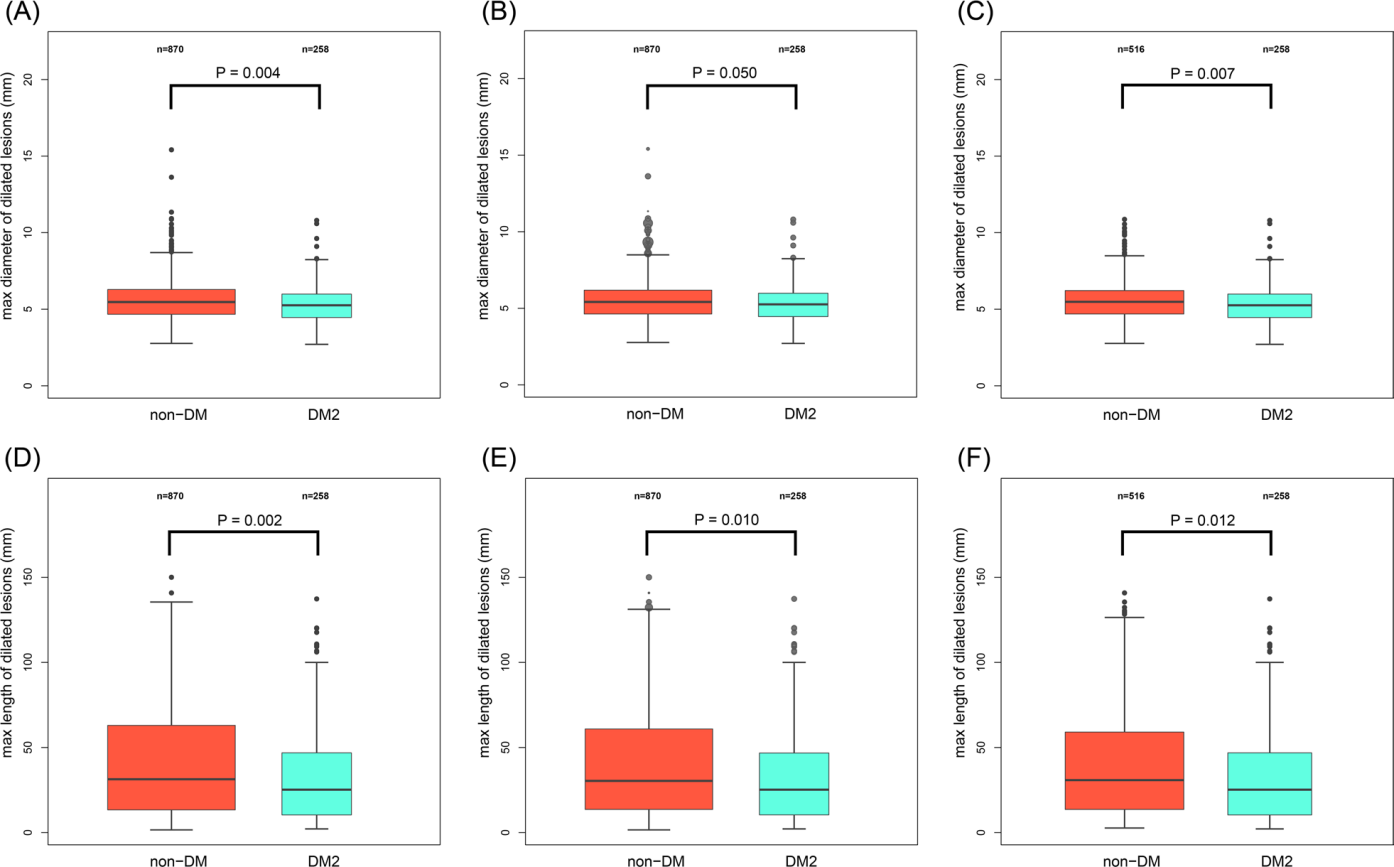
The primary endpoints of the study. The maximum diameter of dilated lesion in unadjusted samples (**A**), propensity score weighted cohort (**B**) propensity score matched cohort (**C**). The length of dilated lesion in unadjusted samples (**D**), propensity score weighted cohort (**E**) propensity score matched cohort (**F**)

Notably, the maximum diameter of dilated lesion was significantly lower in the DM2 group compared with those without DM (5.26 mm vs. 5.47 mm, P  = 0.004) (Fig. 2A). And the maximum length of the dilated lesion was also significantly lower (25.20 mm vs. 31.34 mm, P  = 0.002) (Fig. 2D). Only 57.0% of the patients in the DM2 group had a maximum diameter  > 5 mm, compared with 66.3% in the non-DM group (P  = 0.007).

### Propensity score analysis and subgroup analysis of the primary endpoint

**Fig. 3:**
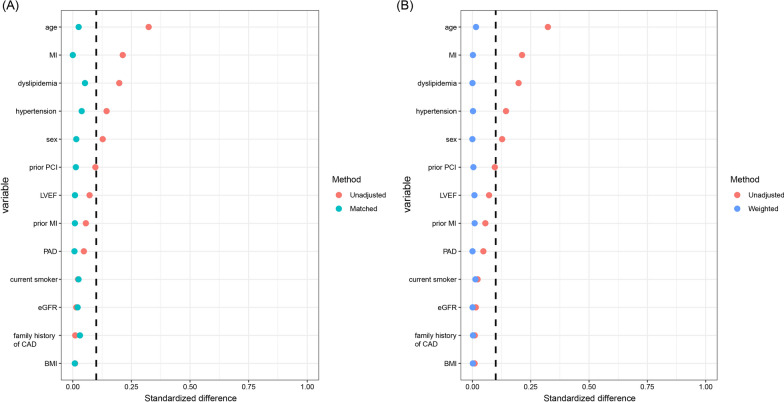
Covariates balance in the study cohort. **A** Standardized difference before and after propensity score weighting. **B** Standardized difference before and after propensity score matching. *BMI *body mass index; *CAD *coronary artery disease; *LVEF *left ventricular ejection fraction; *MI *myocardial infarction; *EGFR *estimated glomerular filtration rate; *PAD *peripheral arterial disease

After adjusting for age, gender, body mass index (BMI), hypertension, dyslipidemia, peripheral arterial disease, smoking status, acute MI, previous MI, previous PCI, family history of CAD, LVEF and eGFR, smaller dilated lesion diameter was consistently observed in PSW cohort and PSM cohort (5.26 mm vs. 5.41 mm, P  = 0.050; 5.26 mm vs. 5.46 mm, P  = 0.007, respectively) (Fig. 2B, C). Shorter lesion length in the DM2 group was also found in the PSW cohort and PSM cohort (25.17 mm vs. 30.17 mm, P  = 0.010; 25.20 mm vs. 30.81 mm, P  = 0.012, respectively) (Fig. 2E, F). The covariates were well balanced and the standardized differences of all the covariates were less than 0.1 after the PS method (Fig. 3). The subgroup analysis of the primary endpoint was shown in Table 3. The trend toward smaller lesion diameter and shorter length were consistently obtained among all subgroups.

**Table 3:** Subgroup analysis of the primary endpoint

Subgroup	Patient no. (DM2 vs. non-DM)	Max diameter (DM2 vs. non-DM)	P value	Max length (DM2 vs. non-DM)	P value
Gender					
Male	208 vs. 743	5.42 [4.55, 6.09] vs. 5.53 [4.74, 6.34]	0.020	29.05 [11.97, 54.51] vs. 33.15 [14.27, 64.75]	0.020
Female	50 vs. 127	4.77 [4.09, 5.57] vs. 5.17 [4.31, 5.83]	0.188	15.62 [6.91, 41.00] vs. 23.19 [9.36, 46.50]	0.076
Age					
> 60	126 vs. 320	5.17 [4.36, 5.86] vs. 5.32 [4.44, 6.11]	0.183	22.88 [9.53, 45.28] vs. 29.21 [13.57, 58.66]	0.045
≤ 60	132 vs. 550	5.35 [4.57, 6.13] vs. 5.60 [4.81, 6.39]	0.029	25.90 [11.51, 52.17] vs. 34.22 [13.29, 64.63]	0.044
BMI (kg/m^2^)					
< 24	54 vs. 199	4.70 [4.20, 5.49] vs. 5.13 [4.47, 6.11]	0.022	15.34 [7.21, 38.40] vs. 23.19 [10.59, 53.10]	0.027
≥ 24 and < 29	129 vs. 462	5.26 [4.44, 5.87] vs. 5.47 [4.64, 6.20]	0.058	25.81 [10.81, 54.40] vs. 28.69 [13.39, 60.57]	0.113
≥ 29	75 vs. 209	5.71 [4.81, 6.23] vs. 5.78 [4.90, 6.45]	0.155	33.36 [13.18, 60.73] vs. 40.14 [19.45, 75.44]	0.034

Values are median [interquartile range]

### Association of glucose-lowering treatment, HbA1c level and the primary endpoint

The subgroup analysis of the DM2 group according to pre-admission metformin use, insulin use and HbA1c level were shown in Table 4. There was no significant difference in the primary endpoint among these subgroups. There was a trend towards higher BMI in patients treated with metformin (27.58 kg/m^2^ vs. 26.84 kg/m^2^, P  = 0.057). After the exclusion of patients taking metformin before admission, DM2 group was still associated with decreased diameter (5.24 mm vs. 5.47 mm, P  = 0.005) and lesion length (24.98 mm vs. 31.34 mm, P  = 0.005), compared with non-DM patients.

**Table 4:** Subgroup analysis in the DM2 group

Subgroup	Patient no.	Max. diameter	P value*	Max. length	P value^#^
Metformin					
Yes	68	5.44 [4.52, 5.92]	0.560	28.73 [10.18, 55.06]	0.766
No	190	5.24 [4.44, 6.07]		24.98 [10.83, 45.28]	
Insulin					
Yes	89	4.96 [4.42, 5.91]	0.306	25.36 [11.73, 43.52]	0.941
No	169	5.41 [4.51, 5.99]		24.59 [10.00, 54.84]	
HbA1c					
> 7.0%	141	5.23 [4.51, 5.88]	0.795	29.18 [11.18, 51.10]	0.382
≤ 7.0%	117	5.28 [4.42, 6.14]		24.02 [10.05, 42.72]	

*Comparation of max. lesion diameter

^#^Comparation of max. lesion length

### Clinical outcomes

**Fig. 4:**
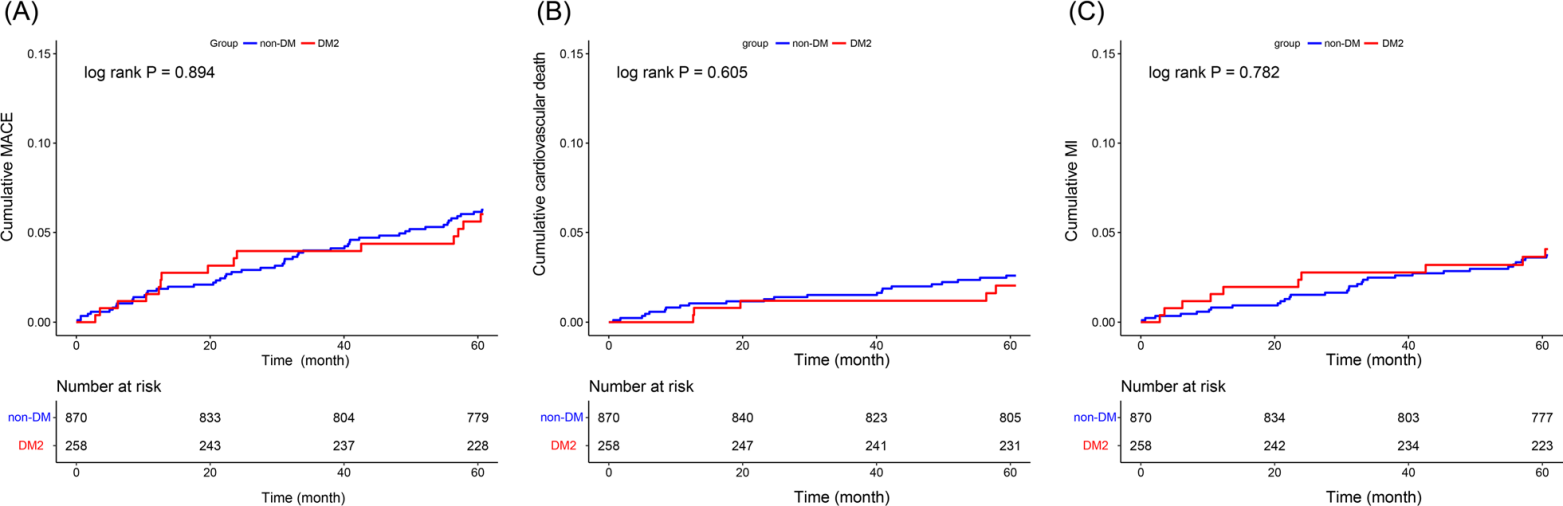
Kaplan–Meier curves showed similar risks of MACE (**A**), cardiovascular death (**B**), and nonfatal MI (**C**) among DM2 group and non-DM group

Follow-up data were available for 95.39% (1076 of 1128) of the study objects at 5 years and a total of 27 cardiovascular deaths and 41 myocardial infarctions occurred. The Kaplan–Meier curves to estimate event rate were shown in Fig. 4. The cumulative risk of MACE (6.02% vs. 6.27%; HR 0.96, 95% CI 0.54–1.71, P  = 0.894), cardiovascular death (2.05% vs. 2.61%; HR 0.78, 95% CI 0.29–2.05, P  = 0.605) and MI (4.07% vs. 3.72%; HR 1.11, 95% CI 0.54–2.26, P  = 0.782) was similar in the DM2 group and the non-DM group. The result of univariable and multivariable Cox regression also suggested similar clinical outcomes among the 2 groups (Table 5).

**Table 5:** Clinical outcomes of the non-DM group and DM group

	Sample size		MACE		
Type of analysis	DM2 group	Non-DM group	Event no.	HR^a^ (95% CI)	P value
Univariable Cox regression	258	870	15 vs. 53	0.96 (0.54–1.71)	0.894
Multivariable Cox regression^b^	258	870	15 vs. 53	0.89 (0.49–1.61)	0.706

^a^Hazard ratio derived from Cox regression model

^b^Multivariable Cox regression adjusted variables including age, gender, BMI, hypertension, dyslipidemia, peripheral arterial disease, current smoker, acute MI, previous PCI, previous MI, family history of CAD, LVEF, eGFR, SYNTAX score, combined CAD, LM ectasia, LAD ectasia, LCX ectasia, RCA ectasia, medications at discharge, concomitant revascularization

### Discussion

In this cohort study of 1128 patients with CAE, we demonstrated that type 2 diabetes mellitus was inversely associated with the maximum diameter of dilated vessels and the maximum length of the dilated lesion. To the best of our knowledge, this is the first study to evaluated the differences of DM2 and non-DM patients in a large cohort of patients with CAE.

CAE was a rare finding in coronary angiography and the reported incidence ranged from 0.2 to 5% [2]. This occurrence rate was 1.07% in the procedure reports of our hospital, which was the national center for cardiovascular disease of China. The baseline and angiographical characteristics were consistent with previous studies [2, 19]. Symptoms varied from symptomless to acute MI. Most patients with CAE were male and combined with atherosclerosis. RCA was the most involved vessel and followed by LAD and LCX.

CAE was considered as a variant of coronary atherosclerosis in the early days [3], but later many studies indicated complex mechanisms of CAE [2] and worse clinical outcomes compared to patients without CAE, including increased risk of death, MI and repeat revascularization [4–6, 20]. Another recent study demonstrated a significantly lower rate of procedural success during primary PCI in patients presenting with CAE and ST-segment elevation myocardial infarction (STEMI), but observed a similar incidence of MACE among patients with and without CAE [21]. Considering the limited sample size of the CAE group in the study (n  = 36), this inconsistent result in long-term clinical outcomes should be interpreted with caution and more data is needed.

Although most of the patients with CAE were combined with atherosclerosis, a relatively low occurrence of diabetes mellitus was found in CAE. The reported incidence of combined diabetes mellitus was 14.7% in a Greece’s cohort [22], 18% in an American cohort [4], 29% in a Japanese cohort [5], and 6.9% in a cohort from the Netherlands [6]. Although the incidence varies greatly, all these studies consistently demonstrated reduced occurrence of diabetes mellitus in patients with CAE, compared to those who didn’t had CAE. A Meta-analysis also suggested a lower incidence of diabetes and it might play a protective role in the development of CAE [23]. The differences in diabetes mellitus occurrence in different cohorts might be a result of different races, inclusion criteria of the studies, diagnostic criteria of DM, and limited sample size in some previous studies. Our study reported 22.9% of patients with CAE were combined with diabetes. As a reference, 32.2% of patients undergoing PCI in our hospital had diabetes mellitus [24]. This was an interesting phenomenon because atherosclerosis was thought to be an important reason for CAE [25] and diabetes was a known risk factor for atherosclerosis.

However, no publication compared the differences between CAE with and without diabetes mellitus by far. This might be because CAE was already rare and there were even fewer patients combined with diabetes. This cohort study, which was comprised of 1128 patients with CAE, demonstrated several differences among patients with and without DM.

Patients in the DM2 group were significantly older and tended to be associated with higher BMI, more hypertension and dyslipidemia. In addition, the DM2 group was combined with more coronary artery disease in coronary angiography evaluation and higher SYNTAX scores were observed, suggesting a higher atherosclerotic burden. This was unsurprising as diabetes mellitus was part of metabolic syndrome [26] and an undisputed risk factor for coronary artery disease. Notably, a shorter length of the dilated lesion and a slightly but significant lower diameter of the dilated vessels were found in DM2 group using QCA analysis. This phenomenon was further confirmed in propensity score weighted and matched analysis. In terms of dilated lesions, patients with diabetes were more focal and relatively less severe. This might partly explain the fewer presence of acute MI in the non-DM group, as diffuse dilation was associated with MI in CAE [27].

Some potential mechanisms could explain the inverse association between DM and dilated lesion extent. Firstly, there was evidence that diabetes impaired compensatory arterial enlargement during the atherosclerotic process and promoted negative arterial wall remodeling [28, 29]. Secondly, matrix metalloproteinase (MMP) was down-regulated in vascular smooth muscle cells and monocytes in diabetes [30, 31], while MMPs were important in the development of coronary aneurysms and transgenic expression of MMP-2 induced coronary artery ectasia in mice models [32]. Increased proteolysis of extracellular matrix proteins is the major pathophysiologic process that leads to ectasia [33], and MMPs played an important role in enzymatic degradation of the extracellular matrix [34]. A study found that both MMP-2 knocked-out mice and MMP-9 knocked-out mice failed to develop abdominal aortic aneurysms (AAA) in a AAA mouse model, indicating that MMPs played a key role in aneurysmal diseases. Thus, it is reasonable to assume that diabetes reduces CAE diameter and extent by down-regulating MMP activity. Thirdly, the inverse association of diabetes mellitus and abdominal aortic aneurysms was also reported, as well as thoracic aortic aneurysms [9]. A recent study demonstrated that patients with diabetes have more than a 35% reduction in the median growth rates of abdominal aortic aneurysms [35]. Considering the association between CAE and aortic aneurysms and the potential common mechanisms [7, 8], the current understanding of diabetes and aortic aneurysm might be helpful. Like in humans, DM showed protective effect on aneurysm development and decreased calcium phosphate-induced aneurysm formation in KK-Ay mice [36]. The mechanistic effects of diabetes on fewer aortic aneurysm and smaller aortic diameter included extracellular matrix remodeling, increase of glycation and advanced glycation end products, decrease of macrophage infiltration in the vascular walls, activation of the TGF-b signaling pathway, and modulation of vascular smooth muscle cells homeostasis [10]. The consistency in lower occurrences of diabetes suggested potential common mechanisms of CAE and aortic aneurysm, as well as the importance of DM’s effect on the pathophysiological process of CAE. However, due to the lack of direct research on CAE and DM, detailed mechanisms of the effect of diabetes on CAE remained future studies.

Metformin use was an important factor and might affect the diameter of aneurysms. Many studies indicated that metformin is associated with a reduction in both growth and clinically relevant events in people with abdominal aortic aneurysm (AAA) [37, 38]. After the exclusion of patients taking metformin, DM is still associated with smaller diameter and shorter length, which further supported the inverse association between DM and CAE lesion diameter and length. However, the current study did not find a negative association between metformin use and lesion diameter or length. Instead, metformin-treated patients appeared to have larger diameters but without statistical significance. Due to the following concerns, the current study doesn’t have enough power to assess the effect of metformin on CAE. Firstly, the sample size of patients taking metformin (n  = 68) is too small. Secondly, the baseline characteristics of patients treated with and without metformin are unbalanced. For example, patients treated with metformin seemed to have a higher BMI, while BMI appeared to be a risk factor of larger diameter. The severity of diabetes is also difficult to quantify. Such confounding factors makes it difficult to assess the effect of metformin use and lesion diameter and length directly in this study. Thirdly, the information of medications was obtained from the electronic medical records, and some detailed information, such like the duration of metformin use, was not available. Fourthly, studies in AAA mainly focused on the growth of the aneurysms, and observed that metformin use is associated with slower growth while the current study reported diameters at diagnosis of CAE. A prospective study specifically designed to address this question in the future would be necessary and valuable.

Despite significant differences in baseline and angiographical characteristics were found in this study, the 5-year MACE risk was similar among DM2 patients and non-DM patients, as well as risks of cardiovascular death and MI. One possible hypothesis was that the seemingly protective effect on the epidemiology of CAE and lesion dilation severity was not strong enough to reduce cardiovascular death and MI. Another reason was that diabetes itself was a risk factor for MACE, which might neutralize the protective effect in CAE. Our recent published article indicated that max diameter  > 5 mm and diffuse CAE (lesion length  > 1/3 of the vessels) was independent predictors of MACE in patients with CAE [39]. Thus, although diabetes is a traditional risk factor for cardiovascular adverse events, it also mitigated the angiographic anatomic risk factors in patients with CAE. This might explain the similar survival between the DM2 group and the non-DM patients. Heterogeneous results were found among studies in DM and outcomes in patients with abdominal aortic aneurysm, some reports increased mortality while others showing no difference or decreased mortality [10]. In this regard, similar survival among DM and non-DM patients in this study is consistent with some previous studies in AAA. In general, the current study emphasized a similar risk of adverse cardiovascular events in DM2 and non-DM patients, although the dilated lesion on coronary angiography seemed more focal and relatively less severe in patients with DM2.

### Study limitation

There are several limitations that should be acknowledged. Firstly, the limitation of an observational cohort study in a single center must be recognized. Secondly, the sample size was relatively small, which was a result of the rarity of CAE and even fewer patients combined with diabetes. Other potential differences between the DM2 group and non-DM group might not be detected due to the weakened statistical efficiency of limited sample size. Thirdly, all the diabetic patients in this study were type 2 DM thus the effect of type 1 DM in CAE remained unclear. Finally, whether there was a growth of dilated lesion in CAE during long-term follow-up and the effect of DM on the growth was not studied. Future studies of dynamic monitoring of coronary angiography and researches in mechanisms are needed.

## Conclusions

Compared to non-DM patients, patients with CAE combined with type 2 diabetes were associated with a smaller diameter and a shorter length of the dilated lesions, suggesting an important effect of DM on the pathophysiological process of CAE. A similar risk of MACE was found in 5-year clinical outcomes among patients with and without diabetes.

## Supplementary Information

**Additional file 1: Table S1.** Coronary artery reference diameter of 231 age-sex-matched angiographically normal subjects.

## Data Availability

The datasets analyzed during the current study are not publicly available due to privacy and ethical restrictions but are available from the corresponding author on reasonable request.

## Abbreviations

CAD: Coronary artery disease
CAE: Coronary artery ectasia
DM: Diabetes mellitus
PS: Propensity score
QCA: Quantitative coronary angiography
