# Supplementary material for: Effect of type 2 diabetes on coronary artery ectasia: smaller lesion diameter and shorter lesion length but similar adverse cardiovascular events
Source: Cardiovasc Diabetol. 2022 Jan 19;21:9. doi: 10.1186/s12933-022-01444-5 (PMC8772080; doi:10.1186/s12933-022-01444-5)
Supplement: Supplementary file 1 — Additional file 1: Table S1. Coronary artery reference diameter of 231 age-sex-matched angiographically normal subjects. [file 12933_2022_1444_MOESM1_ESM.docx]

**SUPPLEMENTARY MATERIAL**

**Table S1. Coronary artery reference diameter of 231 age-sex-matched angiographically normal subjects.**

| Coronary artery segment | reference diameter (mm) |
| --- | --- |
| Right coronary artery |  |
| Proximal | 3.7±0.6 |
| Middle | 3.1±0.5 |
| Distal | 2.8±0.5 |
| Posterior descending | 1.7±0.4 |
| Posterolateral branch | 1.9±0.4 |
| Left anterior descending artery |  |
| Proximal | 3.2±0.6 |
| Middle | 2.8±0.5 |
| Distal | 2.0±0.4 |
| Left circumflex artery |  |
| Proximal | 3.1±0.6 |
| Distal | 2.4±0.4 |
| Obtuse marginal | 1.9±0.5 |
| Left main | 4.4±0.6 |

Values are mean ± SD.
